# Paediatric anaphylaxis in South Africa

**DOI:** 10.1016/j.waojou.2022.100666

**Published:** 2022-09-12

**Authors:** Sa-eeda Chippendale, Kirsten Reichmuth, Margitta Worm, Michael Levin

**Affiliations:** a Department of Paediatrics and Child Health, University of Cape Town, South Africa; b Division of Allergy and Immunology, Department of Dermatology and Allergology, Charité-Universitätsmedizin, Berlin, Germany; c Division of Paediatric Allergology, Department of Paediatrics and Child Health, University of Cape Town, Cape Town, South Africa

**Keywords:** Anaphylaxis, Acute allergic reaction, Paediatric allergy, Food allergy

## Abstract

**Introduction:**

Anaphylaxis is a severe, life-threatening generalized hypersensitivity reaction. While guidelines to reduce the morbidity, risk, and mortality of anaphylaxis are widely available, adherence to these is often suboptimal. We aimed to audit paediatric anaphylaxis at a South African tertiary allergy referral centre, comparing our data to those of the large Network of Severe Allergic Reactions (NORA) registry.

**Methods:**

Children treated for severe allergic reactions between January 2014 and August 2016 were identified for screening using ICD-10 coding of all admissions and discharges, pharmacy records of adrenaline autoinjector dispensing, and additional referrals from the allergy department to the study. Screened participants not meeting the inclusion criteria after preliminary questioning and/or folder review were excluded. Data were collected via a standardized questionnaire using direct interviews, and captured on a local web-based registry.

**Results:**

Of the 156 episodes analysed, >40% were graded as severe and nearly two-thirds of patients were seen for a recurrent episode. Males, younger children, and individuals of mixed-race ethnicity were more frequently affected. Skin and mucosa were most commonly involved, followed by respiratory and gastrointestinal involvement; cardiovascular and other systemic involvement occurred infrequently. Specific IgE assay was the most frequently requested test. Food-related triggers (peanut, hen's egg, fish, cashew nuts and cows' milk) predominated and decreased with age. Anaphylaxis was strongly correlated with atopic conditions. While prophylactic measures were almost universally instituted, adrenaline was rarely used, by both lay persons and healthcare professionals. Hospital admissions were infrequent, and no deaths were recorded.

**Conclusion:**

Management of anaphylaxis can be improved. Specifically, the use of adrenaline prior to hospital arrival remains suboptimal. Ongoing education and training of patients, parents, teachers, and healthcare workers is identified as an area requiring intensification.

## Introduction

Anaphylaxis is “a severe, life-threatening generalized or systemic hypersensitivity reaction”^1^ graded according to organ systems involved and reaction severity.^2^ Guidelines of the World Allergy Organization^3^ and the local Allergy Society of South Africa^4^^,^^5^ promote the following management principles:

• Diagnosis of anaphylaxis is clinical, based on the recognition of characteristic symptoms and signs following exposure to a likely or known trigger. These include respiratory compromise, reduced blood pressure, signs of end-organ dysfunction, involvement of skin and mucosal tissue, and/or severe gastro-intestinal upset.
• Laboratory testing plays a supporting role, especially in ruling out differential diagnoses.
• Triggers vary by age and geography, and over time.
• Patient-specific risk factors, individual co-factors, and concomitant medication use can impact the incidence and severity of anaphylactic episodes.
• Swift emergency management is vital, and frequently rehearsed emergency unit protocols are advocated. Intramuscular adrenaline is first-line treatment,^6^ with repeat dosing as required. Removal of the trigger, calling for help, and supine positioning of the patient is advised. Oxygen and airway management, intravenous access and fluid resuscitation, and cardiopulmonary resuscitation need to be instituted if necessary. Antihistamines, glucocorticoids, inhaled short-acting β_2_-agonists (SABA), inhaled adrenaline, and glucagon are second-line agents of choice.
• Close, frequent, preferably continuous monitoring is recommended, and for a prolonged period (≥12 h) in individuals at risk for biphasic reactions, which include those with severe reactions, and where multiple doses of adrenaline were required.
• Individualized long-term management with prevention of recurrence is emphasized. This should include educating patients and parents about trigger avoidance, discussing and issuing an emergency action plan, prescribing an adrenaline auto-injector (or equivalent) and arranging for medical identification bracelets or tags before discharge. Follow-up with a physician or preferably an allergist/immunologist is strongly advocated for trigger identification, optimization of co-morbid medical management, comprehensive risk assessment, individualized risk-reduction strategies, ongoing education and training, and consideration of immune modulation therapy. Dietician and psychologist referrals are also recommended, for assistance with dietary adjustments if needed, and to optimize compliance and holistic care.
• The need for further anaphylaxis studies and efforts at global partnerships is strongly advised.

The extent of adherence to these management principles is postulated to affect patient morbidity and risk. Although research in this area is challenging, possibly due to under-reporting and low quality of captured data in emergency departments, audits of patient care in anaphylaxis show varying degrees of non-adherence to these principles.^7^ These have largely been attributed to under-recognition and misdiagnosis by medical staff, and to miscoding in the frequently used data capturing methods. Given these challenges, an alternative suggestion to study the characteristics of patients with anaphylaxis is by reviewing adrenaline auto-injection dispensing patterns.^8^

The network of severe allergic reactions (NORA) receives data from allergy centers throughout Europe, including Germany, France, Switzerland, Austria, Spain, Poland, Greece, Bulgaria, Italy, and Ireland. The network collects data from medical records using an online questionnaire, as The European Anaphylaxis Registry.^5^ Standardized information is gathered on incidence, triggering allergens, aggravating factors, demography and medical management.^9^ The aims are to improve the medical management of these patients, facilitate accurate comparisons between centres, highlight public health implications, and examine trends in treatment over time.^10^

At the time of this study, there is remarkably limited data on anaphylaxis from the African continent. Most are case reports and series describing reactions to specific organisms (hydatid,^11^, ^12^, ^13^, ^14^ anisakis,^15^ snakes,^16^^,^^17^ bee stings,^18^^,^^19^ and non-biting midges^20^), plants,^21^ foods^22^ (including specifically cow's milk^23^ and mopane worms^24^), medication (ACE-inhibitors,^25^ snake antivenom,^26^ urografin,^27^ protamine sulphate,^28^ vancomycin,^29^ and BCG vaccination^30^^,^^31^), blood transfusion,^32^ and in certain special circumstances (in otorhinolaryngology,^33^ pregnant women,^34^ and latex in a hospital setting^35^^,^^36^). In African children, anaphylactic shock and severe anaphylaxis has been described during surgery for a hydatid cyst^13^ and after exposure to a trace-amount of cow's milk protein.^23^ A limited cohort study at a South African children's hospital [location masked for blind review] reviewed a series of severe food reactions requiring adrenaline auto-injector prescription.^22^ Other South African studies include a review on the rationale for adrenaline use in anaphylaxis^6^ and a consensus document by the South African Food Allergy Working Group, providing local guidelines for the assessment, investigation and management of food allergies.^5^ There are no register-based African studies.

We aimed to gather data on paediatric anaphylaxis in a referral centre to ascertain our patients’ demographics and culprit allergens, assess management, and appraise risk management strategies. We compare these data with the paediatric data from the NORA register.

## Methods

Patients treated at a South African public children's hospital [location masked for blind review] for severe allergic reactions and anaphylaxis between January 2014 and August 2016 were identified for screening by extracting ICD-10^37^ anaphylaxis codes T78.0, T78.2, T80.5 and T88.6 from the electronic clinical summary system for all admissions and discharges; searching pharmacy records for adrenaline autoinjector dispensing; and new referrals from the staff at the allergy department to the study. These participants were all under and including the age of fourteen, as per the demographic at this facility. Face-to-face interviews with patients and parents were conducted using a standardized questionnaire modified from that initially developed by NORA,^38^ and results confirmed by review of patient records. This was then captured into an electronic registry on the REDCap (Research Electronic Data Capture) web-based application.^39^

Questions included demographics (age at episode, gender, and ethnicity^40^), symptomatology (type, onset, timing, fatality, location, and recurrence), severity based on the Ring and Messner classification system,^2^ diagnostic investigations, previous diagnoses and advice, eliciting triggers, exacerbating factors, concomitant diseases, emergency and preventative treatment, and follow-up.

Patient details were collected for initial recruitment and informed consent, then numerically encrypted and utilized in an anonymous format for database entry and analysis. The REDCap Anaphylaxis Registry was access-restricted, with only approved investigative staff allowed access. This study received approval from the Faculty of Health Sciences Human Research Ethics Committee [ref 510/2015] of a large South African university [location masked for blind review]. Data were analysed using Stata Statistical Software: Release 13, College Station, TX: StataCorp LP.

## Results

### Recruitment

**Fig. 1:**
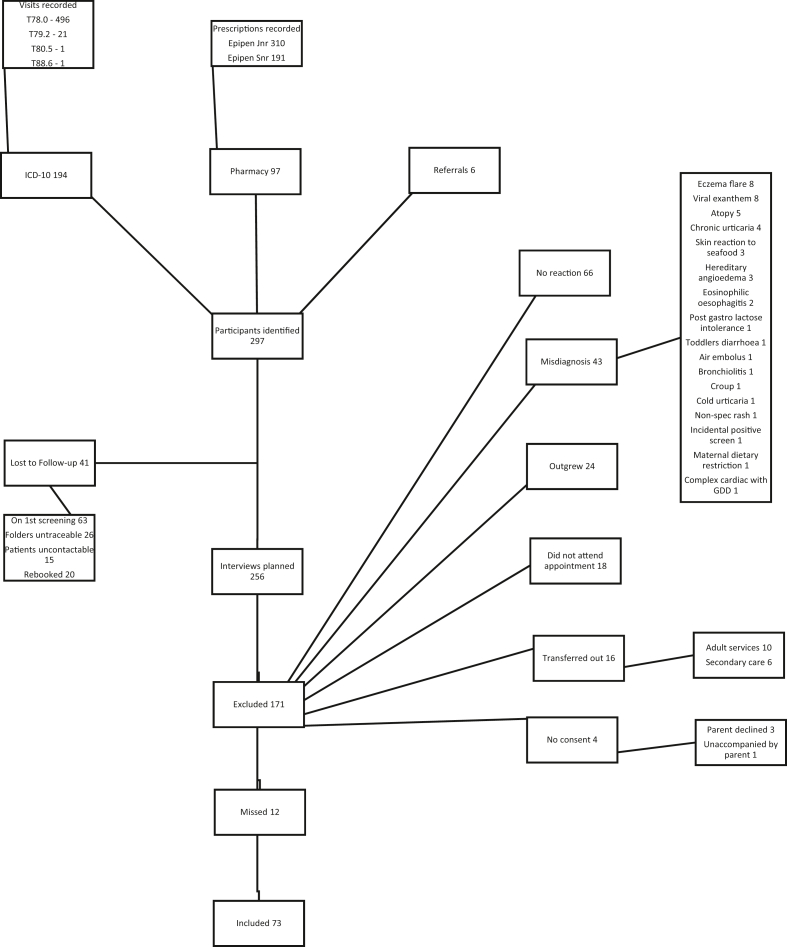
Selection of participants

**Fig. 2:**
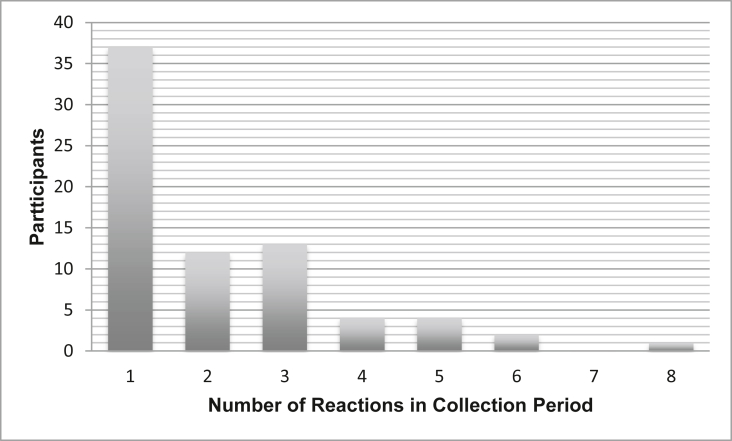
Number of reactions per participant in collection period

All participants were aged 14 and under, as per the demographic at this study facility. Using ICD-10 coding, 519 visits were identified involving 194 patients. Pharmacy records identified 97 patients and direct referrals from the allergy clinic accounted for an additional 6 patients. Of these 297 patients, 41 could not be contacted for further screening. Of the 256 patients screened, 66 reactions did not fall within our data gathering period, 43 patients were miscoded and did not have anaphylaxis, 40 outgrew their diagnosis or were transferred out before our review period, 18 were unavailable for direct interviews, 12 were missed at data collection, 3 parents declined consent, and 1 child was not accompanied by a caregiver able to consent (Fig. 1). Of the 73 patients meeting the inclusion criteria, each child experienced between 1 and 8 reactions in the time specified, amounting to 156 episodes analysed (Fig. 2).

### Demographics and symptomology

**Fig. 3:**
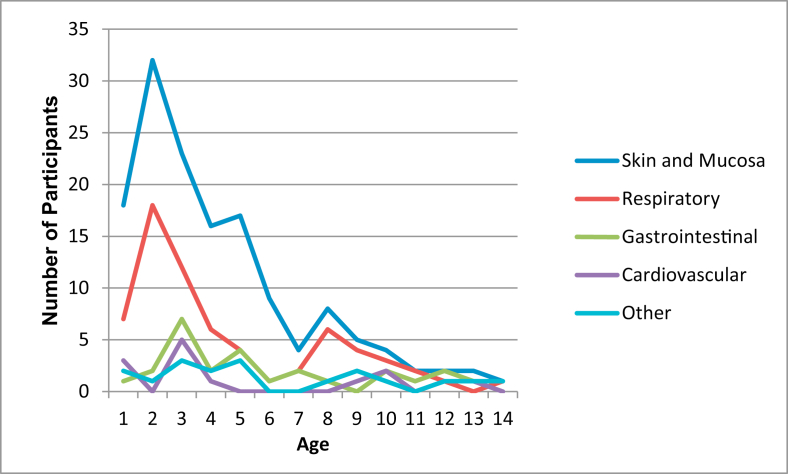
Proportion of systemic manifestations according to age at incident

Males (54.5%), younger participants (78.2% less than 5 years), and individuals of mixed-race ethnicity (82.7%) were more frequently affected. The median age at reaction was 3.0 years (IQR 1.7–5.25). Skin and mucosal surfaces were almost universally involved (143 of 156 reactions; 91.7%), followed by respiratory compromise, with gastro-intestinal upset and cardiovascular symptoms being less common (Fig. 3). Half of the instances recorded were classified as mild (50.0%), with only 8 cases (5.1%) being Grade 2, and the remainder (44.9%) being Grade 3. None of our participants met the Ring and Messner classification of Grade 4. Four episodes (2.6%) were biphasic reactions, all of which occurred 4–12 h after exposure. There were no fatalities during the data collection period. A fifth (20.4%) of all events occurred secondary to a medically supervised allergen challenge in a health care setting, while 65.4% of events happened at home (Table 1).

**Table 1:** Comparisons in patient severity.

	Grade 1	Grade 2	Grade 3	Total
	n (%)	n (%)	n (%)	n (%)
Total	78 (50.0%)	8 (5.1%)	70 (44.9%)	156 (100%)
DEMOGRAPHICS				
*Sex*				
Male	44 (56.4%)	2 (25.0%)	39 (55.7%)	85 (54.5%)
Female	34 (43.6%)	6 (75.0%)	31 (44.3%)	71 (45.5%)
*Age*				
0–5 years	69 (88.5%)	5 (62.5%)	48 (68.6%)	122 (78.2%)
6–14 years	9 (11.5%)	3 (37.5%)	22 (31.4%)	34 (21.8%)
*Ethnicity*				
Black African	7 (9.0%)	2 (25.0%)	11 (15.7%)	20 (12.8%)
White	1 (1.3%)	0	3 (4.3%)	4 (2.6%)
Mixed ethnicity	69 (88.5%)	6 (75.0%)	54 (77.2%)	129 (82.7%)
Indian/Asian	1 (1.3%)	0	2 (2.9%)	3 (1.9%)
SYMPTOMATOLOGY				
*Symptoms*				
Skin and mucosa	78 (100.0%)	7 (87.5%)	58 (82.9%)	143 (91.7%)
Respiratory	4 (5.3%)	5 (62.5%)	58 (82.9%)	67 (42.9%)
Gastro-intestinal	1 (1.3%)	4 (50.0%)	22 (31.4%)	27 (17.3%)
Cardiovascular	0	4 (50.0%)	10 (14.3%)	14 (9.0%)
Other	3 (3.8%)	2 (25.0%)	13 (18.6%)	18 (11.5%)
*Timing*				
Unknown	0	0	3 (4.3%)	3 (1.9%)
0–10 min	59 (75.6%)	6 (75.0%)	56 (80.0%)	121 (77.6%)
11–30 min	7 (9.0%)	0	2 (2.9%)	9 (5.8%)
31–60 min	4 (5.1%)	1 (12.5%)	6 (8.9%)	11 (7.1%)
61–120 min	7 (9.0%)	1 (12.5%)	3 (4.3%)	11 (7.1%)
2–4 h	1 (1.3%)	0	0	1 (0.6%)
>4 h	0	0	0	0
*Biphasic*	1 (1.3%)	1 (12.5%)	2 (2.9%)	4 (2.6%)
*Fatality*	0	0	0	0
*Location*				
Home	49 (62.8%)	6 (75.0%)	47 (67.1%)	102 (65.4%)
Medical practice	20 (25.6%)	2 (25.0%)	10 (14.3%)	32 (20.5%)
Relative/friend's	5 (6.4%)	0	7 (10%)	12 (7.7%)
School/kindergarten	1 (1.3%)	0	3 (4.3%)	4 (2.6%)
Restaurant/takeaway	1 (1.3%)	0	2 (2.9%)	3 (1.9%)
Garden/park	2 (2.6%)	0	0	2 (1.3%)
Unknown	0	0	1 (1.4%)	1 (0.6%)
DIAGNOSTIC TESTING				
*Allergen confirmed (by laboratory testing)*	71 (91.0%)	8 (100.0%)	65 (92.9%)	144 (92.3%)
Before episode	46 (59.0%)	6 (75.0%)	42 (60.0%)	94 (60.3%)
At or after episode	25 (32.0%)	2 (25.0%)	23 (32.9%)	50 (32.0%)
COUNSELLING				
Previously diagnosed	53 (67.9%)	7 (87.5%)	46 (65.7%)	106 (67.9%)
Avoidance advice	52 (66.7%)	6 (75.0%)	45 (64.3%)	103 (66.0%)
Management advice	52 (66.7%)	6 (75.0%)	43 (61.4%)	101 (64.7%)
TRIGGERS *(clinically and by testing)*				
*Known*	74 (94.9%)	8 (100.0%)	66 (94.3%)	148 (94.9%)
Reasonable suspicion	4 (5.1%)	0	1 (1.4%)	5 (3.2%)
*Food: type*				
Peanut	21 (26.7%)	3 (37.5%)	23 (32.9%)	47 (30.1%)
Hen's egg	18 (23.1%)	3 (37.5%)	12 (17.1%)	33 (21.2%)
Fish	9 (11.5%)	0	3 (4.3%)	12 (7.7%)
Cashews	5 (6.4%)	0	7 (10.0%)	12 (7.7%)
Cow's milk	2 (2.6%)	0	7 (10.0%)	9 (5.8%)
Preservative (Na Benz)	5 (6.4%)	0	3 (4.3%)	8 (5.1%)
Hazelnut	3 (3.8%)	0	1 (1.4%)	4 (2.6%)
Shrimp/scampi	2 (2.6%)	0	2 (2.9%)	4 (2.6%)
Sesame	1 (1.3%)	0	2 (2.9%)	3 (1.9%)
Lentil	2 (2.6%)	0	0	2 (1.3%)
Pea	2 (2.6%)	0	0	2 (1.3%)
Mixed nuts	1 (1.3%)	0	1 (1.4%)	2 (1.3%)
Coconut	1 (1.3%)	1 (12.5%)	0	2 (1.3%)
Banana	0	0	2 (2.9%)	2 (1.3%)
Almond	1 (1.3%)	0	0	1 (0.6%)
Bean	0	0	1 (1.4%)	1 (0.6%)
Calamari	0	0	1 (1.4%)	1 (0.6%)
Chocolate	0	0	1 (1.4%)	1 (0.6%)
Colouring agents	1 (1.3%)	0	0	1 (0.6%)
Crayfish	1 (1.3%)	0	0	1 (0.6%)
Legumes	1 (1.3%)	0	0	1 (0.6%)
Pistachio	0	1 (12.5%)	0	1 (0.6%)
*Food: packaging*				
Prepacked	45 (57.7%)	4 (50.0%)	39 (55.7%)	88 (56.4%)
Non-prepacked	33 (42.3%)	3 (37.5%)	28 (40.0%)	64 (41.0%)
*Food: quantity*				
<1 teaspoon	61 (78.2%)	6 (75.0%)	51 (72.9%)	118 (75.6%)
1 teaspoon	14 (17.9.%)	0	10 (14.3%)	24 (15.4%)
1 tablespoon	3 (3.8%)	1 (12.5%)	4 (5.7%)	8 (5.1%)
Unknown	0	0	2 (2.9%)	2 (1.3%)
*Drugs:* Ibuprofen	0	1 (12.5%)	0	1 (0.6%)
EXACERBATING FACTORS				
*Concomitant disease*				
Eczema	73 (93.6.%)	6 (75.0%)	61 (87.1%)	140 (89.7%)
Allergic rhinitis/conjunctivitis	70 (89.7%)	6 (75.0%)	66 (94.3%)	142 (85.3%)
Associated food allergy (separate trigger)	63 (80.8%)	4 (50.0%)	48 (68.6%)	115 (73.7%)
Asthma	49 (62.8%)	1 (12.5%)	56 (80.0%)	106 (67.9%)
Anaemia	7 (9.0%)	2 (25.0%)	7 (10.0%)	16 (10.3%)
Speech delay	5 (6.4%)	2 (25.0%)	4 (5.7%)	11 (7.1%)
Failure to thrive	6 (7.7%)	1 (12.5%)	3 (4.3%)	10 (13.5%)
Gastroeosophageal reflux disease	0	2 (25.0%)	6 (8.6%)	8 (5.1%)
Chronic suppurative otitis media	3 (3.8%)	2 (25.0%)	3 (4.3%)	8 (5.1%)
Papular urticaria	1 (1.3%)	1 (12.5%)	1 (1.4%)	3 (1.9%)
Eosinophilic oesophagitis	2 (2.6%)	0	1 (1.4%)	3 (1.9%)
Chronic constipation	2 (2.6%)	0	1 (1.4%)	3 (1.9%)
ADHD^a^	2 (2.6%)	0	1 (1.4%)	3 (1.9%)
Oppositional defiant disorder	2 (2.6%)	0	1 (1.4%)	3 (1.9%)
Squint	1 (1.3%)	2 (25.0%)	0	3 (1.9%)
Bronchiolitis obliterans	1 (1.3%)	0	1 (1.4%)	2 (1.3%)
Perthe's disease	0	1 (12.5%)	1 (1.4%)	2 (1.3%)
IgA^b^ deficiency	1 (1.3%)	0	0	1 (0.6%)
Epilepsy	0	0	1 (1.4%)	1 (0.6%)
Vestibular migraines	0	1 (12.5%)	0	1 (0.6%)
Autism	0	0	1 (1.4%)	1 (0.6%)
Adjustment disorder	0	1 (12.5%)	0	1 (0.6%)
Conduct disorder	0	0	1 (1.4%)	1 (0.6%)
TREATMENT				
**First line**				
*Attendant*				
Solely lay	42 (53.8%)	3 (37.5%)	27 (38.6%)	72 (46.2%)
Solely professional	24 (30.8%)	3 (37.5%)	21 (30.0%)	48 (30.8%)
Lay then professional	4 (5.1%)	2 (25.0%)	14 (20.0%)	20 (12.8%)
None	8 (10.3%)	0	8 (11.4%)	16 (10.3%)
*Treatment: lay*				
Adrenaline autoinjector	2 (4.3%)	0	9 (22.0%)	11 (12.0%)
Antihistamine	45 (97.8%)	5 (100.0%)	33 (80.5%)	83 (90.2%)
β-2 agonists	0	0	10 (24.4%)	10 (10.9%)
Corticosteroids	0	0	1 (2.4%)	1 (1.1%)
*Treatment: professional*				
Adrenaline IM	1 (3.6%)	2 (40.0%)	9 (25.7%)	12 (17.6%)
Adrenaline IV	0	0	1 (2.9%)	1 (1.5%)
Adrenaline inhaled	0	0	1 (2.9%)	1 (1.5%)
Antihistamine IV	0	0	1 (2.9%)	1 (1.5%)
Antihistamine po	26 (92.9%)	4 (80.0%)	17 (48.9%)	47 (69.1%)
β-2 agonists inhaled	1 (3.6%)	1 (20.0%)	13 (37.1%)	15 (22.1%)
Corticosteroids po	0	1 (20.0%)	0	1 (1.5%)
Oxygen	0	0	7 (20.0%)	7 (4.5%)
Other	2 (7.1%)	1 (20.0%)	10 (28.6%)	13 (19.1%)
*2nd dose adrenaline*	0	0	0	0
**Second line**	1 (1.3%)	3 (37.5%)	15 (21.4%)	19 (12.2%)
*Treatment*				
Corticosteroids po	1 (100.0%)	2 (66.7%)	9 (60.0%)	12 (63.2%)
Antihistamine po	0	2 (66.7%)	5 (33.3%)	7 (36.8%)
β-2 agonists inhaled	0	0	1 (6.7%)	1 (5.3%)
Corticosteroids IV	0	0	1 (6.7%)	1 (5.3%)
**Admission**				
Hospital	2 (2.6%)	2 (25.0%)	17 (24.3%)	21 (13.5%)
ICU^c^	0	0	0	0
PROPHYLAXIS				
*Measures*				
Avoidance counselling	78 (100.0%)	8 (100.0%)	70 (100.0%)	156 (100%)
Drug prescription	78 (100.0%)	8 (100.0%)	70 (100.0%)	156 (100%)
Management plan	78 (100.0%)	8 (100.0%)	70 (100.0%)	156 (100%)
Specific immunotherapy	0	0	0	0
Medic alert bracelet	45 (57.7%)	5 (62.5%)	54 (77.1%)	104 (67.9%)
*Drugs*				
Adrenaline autoinjector	47 (60.3%)	6 (75.0%)	63 (90.0%)	116 (74.4%)
Adrenaline inhaler	0	0	0	0
Antihistamines	78 (100.0%)	8 (100.0%)	70 (100.0%)	156 (100%)
β-2 agonists	49 (62.8%)	1 (12.5%)	63 (90.0%)	113 (72.4%)
Corticosteroids	2 (2.7%)	0	3 (4.3%)	5 (3.2%)

a ADHD, attention deficit hyperactivity disorder.

b IgA, immunoglobulin A.

c ICU, intensive care unit

### Diagnosis and testing

Skin prick testing and specific IgE assays were the mainstays of confirmation of diagnosis, being utilized in 54.5% and 84.0% of reactions, and displaying positive results when tested in 97.6% and 100% of cases respectively. Tryptase levels were requested in only 4.5% of episodes (Table 2).

**Table 2:** South African paediatric anaphylaxis database vs the European anaphylaxis registry's paediatric findings.^9^

	SA study (n = 156)		NORA (n = 1516)		p-value
	n	(%)	n	(%)	
DEMOGRAPHICS					
*Sex*					
Male	85	(54.5)	1021	(67.3)	0.001
Female	71	(45.5)	495	(32.7)	
*Age*					
0–5 years	122	(78.2)	861	(56.8)	0.001
6+ years	34	(21.8)	655	(43.2)	
SYMPTOMATOLOGY					
*Symptoms*					
Skin and mucosa	143	(91.7)	1413	(93.2)	0.483
Respiratory	67	(42.9)	1213	(80.0)	<0.0001
Gastro-intestinal	27	(17.3)	704	(46.4)	<0.0001
Cardiovascular	14	(9.0)	567	(37.4)	<0.0001
Other	18	(11.5)	395	(26.1)	<0.0001
*Timing*					
Unknown	3	(1.9)	0	(0)	0.436
<10 min	121	(77.6)	879	(58.0)	<0.0001
10 min^–1^ h	20	(12.9)	516	(34.0)	<0.0001
>1 h	12	(7.7)	121	(8.0)	0.8952
*Severity*					
Grade 1	78	(50.0)	112	(7.4)	<0.0001
Grade 2	8	(5.1)	631	(41.6)	<0.0001
Grade 3	70	(44.9)	758	(50.0)	0.225
Grade 4	0	(0)	12	(0.8)	0.263
*Biphasic*	4	(2.6)	60	(4.0)	0.388
*Fatality*	0	(0)	4	(0.3)	0.493
*Location*					
Home (own or external)	114	(73.1)	778	(51.3)	<0.0001
Medical practice	32	(20.5)	137	(9.0)	<0.0001
School/kindergarten	4	(2.6)	141	(9.3)	0.005
Restaurant/takeaway	3	(1.9)	58	(3.8)	0.227
Garden/park	2	(1.3)	186	(12.3)	<0.0001
Urban public place	0	(0)	58	(3.8)	0.013
Unknown	1	(0.6)	0	(0)	0.003
*Previous reaction*	101	(64.7)	461	(30.4)	<0.0001
More than 1 previous	61	(39.1)	182	(12.0)	<0.0001
Milder and/or similar	37	(23.7)	303	(20.0)	0.027
More severe	89	(57.1)	155	(10.2)	<0.0001
DIAGNOSTIC TESTING					
*Allergen confirmed*	144	(92.3)	1007	(66.4)	<0.0001
Before episode	94	(60.3)	310	(20.4)	<0.0001
At or after episode	50	(32.0)	697	(46)	<0.001
*Testing done*					
Skin test	85	(54.5)	977	(64.4)	<0.0001
Intradermal test	0	(0)	74	(4.9)	0.005
Provocation test	7	(4.5)	155	(10.2)	0.022
sIgE^a^			773	(51.0)	
RAST^b^	131	(84.0)	279	(18.4)	<0.0001
CAST^c^	10	(6.4)	0	(0)	<0.0001
Tryptase	7	(4.5)	425	(28.0)	<0.0001
*Results positive*					
Skin test	83	(53.2)	861	(56.8)	0.388
Intradermal test	0	(0)	63	(4.2)	0.009
Provocation test	1	(0.6)	134	(8.8)	<0.001
sIgE^a^	0	(0)	723	(47.7)	0.082
RAST^b^	131	(84.0)	250	(16.5)	<0.0001
CAST^c^	10	(6.4)	0	(0)	<0.0001
Tryptase	0	(0)	35	(2.3)	0.056
TRIGGERS					
*Known*	148	(94.9)	1259	(83.0)	<0.0001
Reasonable suspicion	5	(3.2)	204	(13.5)	<0.001
*Food: type*	150	(96.2)	1106	(73.0)	<0.0001
Peanut	47	(30.1)	291	(19.2)	0.001
Hen's egg	33	(21.2)	129	(8.5)	<0.0001
Fish	12	(7.7)	19	(1.3)	<0.0001
Cashews	12	(7.7)	83	(53.2)	<0.0001
Cow's milk	9	(5.8)	133	(8.8)	0.201
Preservative (Na Benz)	8	(5.1)	0	(0)	<0.0001
Other legumes	4	(2.6)	33	(2.2)	0.748
Hazelnut	4	(2.6)	81	(5.3)	0.142
Shrimp/scampi	4	(2.6)	11	(0.7)	0.015
Sesame	3	(1.9)	15	(1.0)	0.301
Other tree nuts	3	(1.9)	26	(1.7)	0.855
Pea	2	(1.3)	12	(0.8)	0.516
Coconut	2	(1.3)	0	(0)	<0.0001
Other fruits	2	(1.3)	26	(1.7)	0.710
Calamari	1	(0.6)	0	(0)	0.003
Chocolate	1	(0.6)	0	(0)	0.003
Colouring agents	1	(0.6)	0	(0)	0.003
Crayfish	1	(0.6)	0	(0)	0.003
Pistachio	1	(0.6)	21	(1.4)	0.692
Walnut	0	(0)	46	(3.0)	0.028
Pine nut	0	(0)	13	(0.9)	0.234
Other tree nuts	0	(0)	26	(1.7)	0.011
Celery	0	(0)	4	(0.3)	0.493
Other vegetables	0	(0)	8	(0.5)	0.376
Wheat	0	(0)	27	(1.8)	0.091
Other cereals	0	(0)	13	(0.9)	0.234
Goat's milk	0	(0)	12	(0.8)	0.262
Other animal products	0	(0)	42	(2.8)	0.034
Soy	0	(0)	12	(0.8)	0.262
Other spices	0	(0)	11	(0.7)	0.295
*Food: packaging*					
Prepacked	88	(56.4)	–		
Non-prepacked	64	(41.0)	–		
*Food: quantity*					
<1 teaspoon	118	(75.6)	–		
1 teaspoon	24	(15.4)	–		
1 tablespoon	8	(5.1)	–		
Unknown	2	(1.3)	–		
*Drugs*	1	(0.6)	52	(3.4)	0.056
Analgesics	1	(0.6)	18	(1.2)	0.502
Cephalosporins	0	(0)	11	(0.7)	0.295
Penicillin	0	(0)	6	(0.4)	0.429
*Insects*					
Yellow jacket	0	(0)	122	(8.0)	<0.001
Bee	0	(0)	105	(6.9)	<0.001
Hornet	0	(0)	10	(0.7)	0.292
*Immunotherapy*	0	(0)	32	(2.1)	0.068
EXACERBATING FACTORS					
*Concomitant disease*					
Allergic rhinitis/conjunctivitis	142	(91.0)	290	(19.1)	<0.0001
Eczema	140	(89.7)	458	(30.2)	<0.0001
Associated food allergy (separate trigger)	115	(73.7)	5	(0.3)	<0.0001
Asthma	106	(67.9)	340	(22.4)	<0.0001
Urticaria	0	(0)	17	(1.1)	0.188
Mastocytosis	0	(0)	2	(0.1)	0.693
Anaemia	16	(10.3)	0	(0)	<0.0001
Speech delay	11	(7.1)	0	(0)	<0.0001
Failure to thrive	10	(6.4)	0	(0)	<0.0001
Gastroeosophageal reflux disease	8	(5.1)	0	(0)	<0.0001
Chronic suppurative otitis media	8	(5.1)	0	(0)	<0.0001
Papular urticaria	3	(1.9)	0	(0)	<0.0001
Eosinophilic oesophagitis	3	(1.9)	0	(0)	<0.0001
Chronic constipation	3	(1.9)	0	(0)	<0.0001
ADHD^d^	3	(1.9)	0	(0)	<0.0001
Oppositional defiant disorder	3	(1.9)	0	(0)	<0.0001
Squint	3	(1.9)	0	(0)	<0.0001
Bronchiolitis obliterans	2	(1.3)	0	(0)	<0.0001
Perthe's disease	2	(1.3)	0	(0)	<0.0001
IgA^e^ deficiency	1	(0.6)	0	(0)	0.003
Epilepsy	1	(0.6)	0	(0)	0.003
Vestibular migraines	1	(0.6)	0	(0)	0.003
Autism	1	(0.6)	0	(0)	0.003
Adjustment disorder	1	(0.6)	0	(0)	0.003
Conduct disorder	1	(0.6)	0	(0)	0.003
*Co-factors*					
Physical exercise	0	(0)	277	(18.3)	<0.0001
Psychological stress	0	(0)	30	(2.0)	0.075
Medication	0	(0)	64	(4.2)	0.007
TREATMENT					
**First line**					
*Attendant*					
Lay	92	(59.0)	464	(30.6)	<0.0001
Professional	68	(43.6)	1014	(66.9)	<0.0001
None	16	(10.3)	454	(29.9)	<0.0001
*Attendant: lay*					
Self-treated	3	(1.9)	20	(1.3)	0.537
Nursery/schoolteacher	1	(0.6)	26	(1.7)	0.297
Other	87	(55.8)	0	(0)	<0.0001
*Attendant: professional*					
Emergency physician	32	(20.5)	382	(25.2)	0.195
Allergy specialist	29	(18.6)	116	(7.7)	<0.0001
Other	7	(4.5)	0	(0)	<0.0001
*Treatment: lay*					
Adrenaline autoinjector	11	(7.1)	52	(3.4)	0.021
[PRESENT BUT NOT USED]	75	(48.1)	71	(4.7)	<0.0001
Antihistamine	83	(53.2)	371	(24.5)	<0.0001
β-2 mimetic	10	(6.4)	135	(8.9)	0.291
Corticosteroids	1	(0.6)	244	(16.1)	<0.0001
*Treatment: professional*					
Adrenaline IM	12	(7.7)	168	(11.1)	0.204
Adrenaline IV	1	(0.6)	55	(3.6)	0.047
Adrenaline inhaled	1	(0.6)	81	(5.3)	0.009
Antihistamine IV	1	(0.6)	413	(27.2)	<0.0001
Antihistamine po	47	(30.1)	367	(24.2)	0.104
β-2 mimetic inhaled	15	(9.6)	235	(15.5)	0.049
Corticosteroids IV	0	(0)	503	(33.2)	<0.0001
Corticosteroids po	1	(0.6)	211	(13.9)	<0.0001
Corticosteroids PR	0	(0)	127	(8.4)	<0.001
Oxygen	7	(4.5)	90	(5.9)	0.475
Fluids	0	(0)	203	(13.4)	<0.0001
Other	13	(8.3)	0	(0)	<0.0001
*2nd Dose Adrenaline*	0	(0)	88	(5.8)	0.002
**Second line**	19	(12.2)	201	(13.3)	0.699
*Treatment*					
Corticosteroids po	12	(7.7)	–		
Antihistamine po	7	(4.5)	–		
β-2 mimetic Inhaled	1	(0.6)	–		
Corticosteroids IV	1	(0.6)	–		
**Admission**					
Hospital	21	(13.5)	301	(19.9)	0.054
ICU^f^	0	(0)	16	(1.1)	0.188
PROPHYLAXIS					
*Measures*					
Avoidance counselling	156	(100)	1389	(91.6)	<0.001
Drug prescription	156	(100)	1399	(92.3)	<0.001
Management plan	156	(100)	1374	(90.6)	<0.001
Specific immunotherapy	0	(0)	174	(11.5)	<0.0001
Medic alert bracelet	104	(67.9)	0	(0)	<0.0001
*Drugs*					
Adrenaline autoinjector	116	(74.4)	1260	(83.1)	0.007
Adrenaline inhaler	0	(0)	0	(0)	
Antihistamines	156	(100)	1371	(90.4)	<0.001
β-2 mimetics	113	(72.4)	503	(33.2)	<0.0001
Corticosteroids	5	(3.2)	1273	(84.0)	<0.0001

a sIgE, specific Immunoglobulin E.

b RAST, radioallergosorbent test.

c CAST, cellular antigent stimulation test.

d ADHD, attention deficit hyperactivity disorder.

e IgA, immunoglobulin A.

f ICU, intensive care unit

### Triggers

**Fig. 4:**
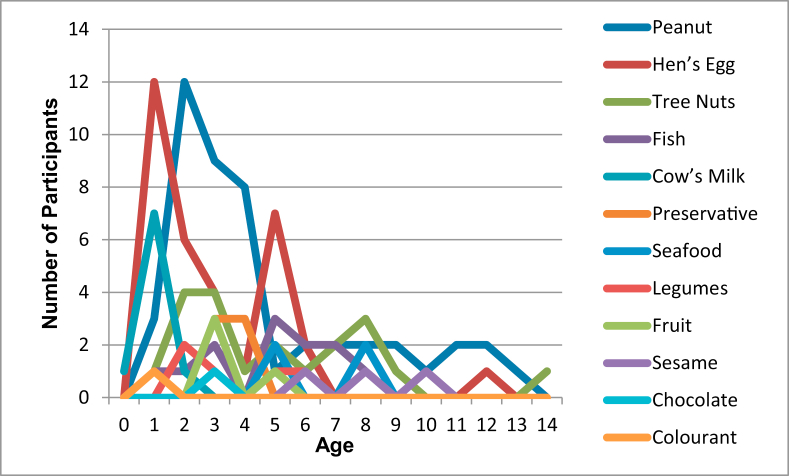
Proportion of triggers by age at incident

On review of patient history and diagnostic testing, three instances (1.9%) were caused by an unknown allergen, one (0.6%) by drugs (ibuprofen), and the remainder by a food-related trigger (Table 1). Cow's milk played a significant role as a trigger in children under 2 years of age, hen's egg in toddlers, and peanuts as a trigger in school-going children (Fig. 4). The majority of food reactions (75.6%) were caused by very small amounts of food ingested, ie, less than one teaspoonful. Of the 88 episodes where children had a reaction to pre-packed foods, the trigger was noted in the product name or listed in the ingredients in 60 (68.2%) cases and in the “may contain” advice box in 3 (3.4%) cases. For 25 (28.4%) cases, the labelling could not be recalled. Of the 64 children who had reactions to non-pre-packed foods, 39 (60.9%) had reactions to homemade food, 20 (31.3%) to catered food, 3 (4.7%) to food from a fishmonger, and 2 (3.1%) to food from a bakery.

### Associated conditions

Atopic conditions were strongly associated with severe allergic reactions, with 89.7% of reactions occurring in patients known with atopic eczema, 85.3% in patients with allergic rhinoconjunctivitis and 67.9% in patients with asthma (Table 1). In 115 (73.7%) of instances, the patients had an associated food allergy to a second food. Additional co-morbid conditions noted with lower frequency include other allergic/immune (eosinophilic oesophagitis, papular urticaria, IgA deficiency), nutritional (anaemia and failure to thrive), respiratory (bronchiolitis obliterans), gastrointestinal (gastroesophageal reflux disease and chronic constipation), neurological (squint, epilepsy, vestibular migraines), neurodevelopmental/neuropsychiatric (speech delay, autism, attention deficit hyperactivity disorder, adjustment disorder, conduct disorder, oppositional defiant disorder), and orthopaedic (Perthes disease). No co-factors were identified in our sample population.

### Management

In total, 10.3% (16 of 156) of instances went untreated. Of those treated, a lay person was the first responder in 92 (59.0%) cases, 20 (21.7%) of these then also seeking professional help. Of these lay responders, 87 (94.6% of 92) were managed by a family member, usually the parent; three (3.3%) were self-managed; and one (1.1%) was managed by a nursery school teacher. Antihistamines were used as first-line treatment by most (83 of the 92; 90.2%) lay-persons, while the use of adrenaline auto-injectors and inhaled SABA was rare: 11 (12.0%) and 10 (10.9%) respectively (Table 1, Table 2).

Initial management was provided by a professional in 68 (53.6%) cases, 20 (29.4%) of which were preceded by lay person treatment. When professionals were involved in emergency care, this entailed an emergency doctor or general practitioner in 38 (55.9% of 68) instances and an allergy specialist in 29 (42.6% of 68) cases. All allergen challenges (32 of 156; 20.5%) were managed by the supervising allergy specialists. Adrenaline was administered as first-line care by the attending health professionals intramuscularly in 12 cases (17.6%), intravenously once (1.5%), as an inhalant once (1.5%), and not at all in 54 cases (79.4%). Oral antihistamines (47; 69.1%) and inhaled SABA (15; 22.1%) were the most frequently used agents by health care professionals, with intravenous antihistamines and oral corticosteroid administered once (1.5%) each (Table 1). In nineteen instances (12.2%), patients were administered additional treatment after initial stabilization, mostly oral corticosteroids (12; 63.2% of 19) or a second dose of antihistamine (7; 36.8%). Twenty-one patients (13.5%) were admitted to hospital, but none required intensive care.

### Prophylaxis

Preventive measures were instituted before and after the recorded reactions to varying degrees (Table 3). Most patients/caregivers received counselling regarding the condition and all received emergency medication and training. Of the 73 participants in this study, 43 (58.9%) were assisted with Medic Alert application. Of these, 3 (4.1%) were still awaiting delivery, 2 (2.7%) had their bracelet/tag stolen, 3 (4.1%) had lost theirs, one (1.4%) was using a second one, and in 2 cases (2.7%) the child refused to wear the bracelet/tag. Oral antihistamines were prescribed in all patients, adrenaline autoinjectors in 116 (74.4%), and inhaled SABA in 113 (72.4%).

**Table 3:** Timing of prophylactic measures

	Prior to reaction		Prior to discharge		During primary care follow-up	During specialist follow-up		Nil	
	n	(%)	n	(%)		n	(%)	n	(%)
Counselling about avoidance of trigger	103	(66.0%)	62	(39.7%)	–	153	(98.1%)	3	(1.9%)
Prescription of emergency drugs	124	(79.5%)	63	(40.4%)	–	156	(100.0%)	0	
Adrenaline autoinjector	84	(53.8%)	24	(15.4%)	–	116	(74.5%)	39	(25.0%)
Adrenaline inhaler	–		–		–	–		–	
Antihistamines	139	(89.1%)	55	(35.3%)	–	15	(9.6%)	0	
β-2 agonists	96	(61.5%)	23	(14.7%)	–	113	(72.4%)	43	(27.6%)
Corticosteroids	2	(1.3%)	2	(1.3%)	–	3	(1.9%)	151	(96.8%)
Training in emergency management plan	117	(75.0%)	48	(30.8%)	–	156	(100.0%)	0	
Specific immunotherapy	–		–		–	–		–	
Medic-alert identification	77	(49.4%)	0		–	27	(17.3%)	52	(33.3%)

### Recurrence

Of the 156 episodes, 101 (64.7%) were preceded by a previous reaction to the same allergen. Of these, 36 (35.6%) had a single preceding event, 28 (27.7%) had 2, 7 (6.9%) had 3, 26 (25.7%) had more than 3, and in 4 instances (3.9%) patients could not recall the number of previous reactions. Of the preceding episodes, 89 (88.1%) were severe, with 37 (36.6%) recalled as being milder than the recorded event. The organ systems most commonly involved in previous reactions were the skin (97; 96.0%) and respiratory (73; 72.3%) system, with gastrointestinal (18; 17.8%) and cardiovascular (5; 5.0%) involvement being rarer.

In 106 episodes, patients were aware of or suspected an underlying allergy to the offending agent. This is in excess of the number of participants who experienced a previous reaction to the current allergen (101) as some children had the offending allergen identified as a potential trigger on previous investigation, after an event following exposure to a different allergen, before the episode in question. Prior diagnosis was made by a general practitioner or emergency physician in 22 instances (20.8%), an allergy specialist in 77 (72.6%), and was self-diagnosed by the parent in the remaining seven (6.6%).

In patients who already had prophylactic medication prescribed to them before an acute allergic reaction occurred, eg., anaphylaxis or a concomitant disease, antihistamines were used most frequently in the emergency situation. Adrenaline and SABA were used less often, and corticosteroids were not used at all (Table 4).

**Table 4:** Patterns of medication use in an acute allergic reaction, in patients with previously prescribed medication (including for concomitant disease)

	Prescribed and used		Prescribed, available, but not used		Prescribed, but not available		Total
	N	(%)	n	(%)	n	(%)	
Adrenaline	32	(38.1%)	40	(47.6%)	12	(14.3%)	84
SABA	35	(36.5%)	60	(62.5%)	1	(1.0%)	96
Antihistamine	114	(82.0%)	19	(13.7%)	6	(4.3%)	139
Corticosteroid	0		2	(100%)	0		2

## Discussion

In keeping with international studies,^10^^,^^41^ severe allergic reactions were more common in males and children in the younger age groups. For comparison, we contrasted our findings with the European Anaphylaxis Registry, that reviewed 1970 children from 90 centres in ten countries (Table 2). In our data, the majority of cases occurred within the first few years of life, although there were smaller peaks at ages five and eight, possibly correlating to prolonged trigger avoidance, followed by self-induced or doctor-led allergen challenges.

We added self-defined ethnicity as per the latest StatsSA census classification.^40^ Ethnicity was not included in the European study, but a systematic review of the literature^41^ shows African ethnicity as a potential risk factor for fatal anaphylactic episodes, with limited data on the effect of ethnicity on non-fatal episodes. We unexpectedly had disproportionately more participants who self-classified as mixed ethnicity in this study (82.7%). This distribution could only partially be explained by socio-economic disparities and differing health seeking behaviour between socio-demographic groups in Cape Town, as this mixed-race proportion in our study (82.7%) was not congruent to the spectrum of patients seeking health care at the hospital for other medical conditions (52.0%, p < 0.0001), or the mixed-race proportion of the ethic profile of the Western Cape (48.8%,^42^ p < 0.0001) and South Africa (8.9%,^43^ p < 0.0001). There was no significant effect of ethnicity on severity (p = 0.428).

The pattern of systemic involvement is in keeping with global trends. The severity of distribution differs from that of the European Anaphylaxis Registry,^9^ with more grade 1 and 3 reactions observed here than grade 2 and 3 as seen in the data of our counterparts. This could be accounted for by a lower threshold for inclusion locally due to our rigorous recruitment process, rather than requiring a primary care doctor to report to a central research agency as per the NORA study. The timing between exposure and reaction was less than 10 min in most of our patients, similar to that seen in international studies. We recorded proportionately less biphasic reactions, with our patterns occurring at 4–12 h after exposure, instead of the more than 12 h in the NORA cohort. A comparatively larger proportion of our reactions happened at home (Table 2).

Diagnostic testing seemed to be used appropriately in our resource-limited setting, with the majority of triggers identified, an almost universal positive pick-up rate by the tests utilized, and low rates of multiple allergen screens and negative results. Most diagnoses were made at follow-up with the allergy sub-specialist, in identical proportions to the above studies. Two-thirds of patients were noted to be allergic to the offending allergen before the recorded event, also similar to the European data. Almost all were advised regarding avoidance of the trigger and emergency managements, but the practical effectiveness of this needs to be addressed in view of the relatively high rate of recurrent reactions and non-use of the prescribed medications in the emergency situation.

With our comparatively smaller sample size, no reactions were associated with insects and antibiotics, or with immunotherapy. In the European study, peanuts, cows' milk and hen's eggs predominate as food triggers, decreasing with age. Our trend is similar, with the addition of fish and tree nuts (particularly cashew nuts) playing a larger role, potentially due to our increased incidence of ingestion of the former, and possible decreased awareness of the latter.

The association of allergic reactions in our population to atopic disorders and food hypersensitivity to a second trigger mirrors the European trend, but at a more than three-fold increase in rate: The incidence of eczema is 89.7% in our participants (compared to 26.3% in the European database), allergic rhinitis 85.3% (vs 21.2%), asthma 67.9% (vs 22.9%), and food allergies to a different agent 73.7% (vs 0.5%). This can only partly be explained by the tertiary setting of patient sampling and suggests further avenues for investigative research. The co-morbid anaemia and failure to thrive may be caused by parental-led highly restrictive diets in subjects with multiple food allergy. These prevalence rates, along with those of the neuro-developmental and psychiatric conditions, are difficult to interpret without a baseline population comparison. No other major exacerbating factors were diagnosed.

A large proportion of all episodes in our study were solely managed by a lay person, usually a parent, occasionally self-administered, and rarely by a teacher. Internationally, a comparatively larger proportion was treated by professionals. This may be due to the local under-recognition of the severity of the underlying condition, different health seeking behaviours and access to health care in our setting, and low public and school awareness of anaphylaxis and its management. The majority of first medical attenders were non-allergy specialists. Adrenaline was rarely administered, by lay responders and professionals. This calls for intensification of education to schools and emergency department staff. Fewer of our participants were admitted to hospital, either due to the comparatively higher proportion of less severe reactions reviewed or to lack of awareness of the need for hospital admission after anaphylactic episodes to observe for and treat biphasic reactions.^7^ Application of preventative measures needs improvement, particularly the issuing of adrenaline autoinjectors in the severely affected group of participants and referral for Medic Alert identification. Education to parents and patients also requires intensification, as despite a high rate of counselling and training, 10% of reactions went untreated in the acute situation, half of which were severe.

### Strengths and limitations

This is the first African series of anaphylaxis patterns, allowing for comparisons with global studies. The recruitment process was systematic, as opposed to an opportunistic multi-centred approach, accounting for a lower threshold for inclusion locally. A tertiary setting for this study was appropriate, in keeping with recommended follow-up guidelines, but the potential for missing mismanaged unreferred potential participants exists. Analysis of trends over time was not possible due to the short study period. The study was also based at a single centre, resulting in a limited sample size, and in a tertiary subspecialist referral setting which would likely reflect improved management compared to treatment at primary care. In addition, some potential participants were lost to follow-up or excluded for other reasons. (Fig. 1).

The reliance on parental memory might be biased, but is ameliorated by our review of the associated hospital records.

One death occurred during our collection period, but could not be included for analysis. This was due to a study design limitation: our study was approved by the ethics committee for face-to-face interviews at routine follow-up, with doctors’ notes as retrospective support. The European Anaphylaxis Registry entailed taking consent at a first visit and collecting data from folder reviews pro- and retrospectively for the study time demarcated, which allowed inclusion of deaths.

## Conclusion

This is the first comprehensive descriptive review of local anaphylactic trends. In comparison to similarly conducted European studies, certain discrepancies would benefit from further investigation: particularly the propensity for allergic reactions in the mixed ethnicity population, as well as our much higher rate of association with other allergic conditions compared to international patterns. An analysis of our baseline comorbid disorders would also assist in putting this review in context, and an investigation into barriers to care could assist with patient care. This further serves as a motivation for more locally-based, internationally-standardized anaphylaxis registries and research. Intensification of educational efforts to patients, parents, schools, and medical teams is strongly advised.

## Abbreviations

ICD10, International Statistical Classification of Diseases and Related Health Problems, 10th edition; NORA, The network of severe allergic reactions; RedCAP, Research Electronic Data Capture; SABA, Short-acting β2-agonist.

## Financial support

University of Cape Town Paediatric Department Research Award, 2018.

University of Cape Town Dyssell Fund Award, 2019.

## Availability of data and materials

All data is available for secondary analysis.

## Author contributions

All authors contributed equally to conception, design, drafting and approval of this study.

## Ethics statement

Approved by the University of Cape Town's Faculty of Health Sciences Human Research Ethics Committee [ref 510/2015].

## Authors’ consent for publication

All authors have given consent for publication.

## Confirmation of original work

This work has not previously been published and is not under consideration for publication elsewhere.

## Declaration of competing interest

None to declare.
